# [ ^99m^ Tc]Tc-Pyrophosphate Scintigraphy for Cardiac Amyloidosis: Evaluation of Scintigraphy Findings and Their Prognostic Value

**DOI:** 10.1055/s-0045-1813662

**Published:** 2025-11-23

**Authors:** Elife Akgün, Ümit Aksu, Arda Güler, Gamze Babur Güler, Meryem Kaya, Burcu Esen Akkaş

**Affiliations:** 1 Department of Nuclear Medicine, University of Health Sciences Türkiye, İstanbul Training and Research Hospital, Istanbul, Türkiye; 2 Department of Nuclear Medicine, University of Health Sciences Türkiye, Başakşehir Çam and Sakura City Hospital, Istanbul, Türkiye; 3 Department of Cardiology, University of Health Sciences Türkiye, Mehmet Akif Ersoy Thoracic and Cardiovascular Surgery Training and Research Hospital, Istanbul, Türkiye

**Keywords:** ATTR, AL, cardiac amyloidosis, PYP, scintigraphy, prognosis

## Abstract

**Background:**

Association of [ ^99m^ Tc]Tc-pyrophosphate (PYP) uptake with survival of cardiac amyloidosis (CA) is still unknown. This study aimed to describe the scintigraphic findings of CA suspected cases and determine the prognostic value of scintigraphy.

**Methods:**

Between September 2020 and December 2023, all [ ^99m^ Tc]Tc-PYP scintigraphy images of CA suspected cases were evaluated retrospectively. The scintigraphy images were obtained 1 and 3 hours after intravenous injection of 740 MBq [ ^99m^ Tc]Tc-PYP. The quantitative and semiquantitative results of scintigraphy were analyzed, and their correlation with survival was evaluated.

**Results:**

Among the 268 cases (147 females, 121 males; median age: 65), 12 (4.5%) were diagnosed with transthyretin (ATTR) CA and 19 (7.1%) with light chain (AL) CA. The median follow-up time was 386 days. ATTR CA cases exhibited significantly higher [ ^99m^ Tc]Tc-PYP uptake than AL CA and non-CA cases. The 1- and 3-hour heart-to-contralateral lung ratios were positively highly correlated with each other, and SPECT/CT (single-photon computed tomography/computed tomography) imaging reduced equivocal results from 82 to 37%. Eight of the AL CA cases (42.1%), 1 ATTR CA case (8.3%), and 15 of non-CA cases (6%) died on follow-up. Median survival was 22 days in ATTR CA, 201 days in AL CA, and 105 days in non-CA deceased cases. Survival analysis indicated significantly lower survival in non-CA cases compared with ATTR CA and AL CA.

**Conclusion:**

[ ^99m^ Tc]Tc-PYP scintigraphy is highly sensitive and specific for detecting ATTR CA and differentiating from AL CA. Even in the absence of a diagnosis of CA, the fact that survival may be short in cases with high uptake on scintigraphy should be considered in the follow-up of these cases.

## Introduction

Misfolded amyloid proteins formed from light chain (AL) or transthyretin (ATTR) accumulation in the myocardium cause cardiac amyloidosis (CA). These depositions result in thickening of different parts of the heart and affect heart function. It is one of the reasons for restrictive cardiomyopathy. Although amyloidosis is a multisystem disease, generally, cardiac involvement determines the prognosis.

AL amyloidosis is a rare disease with an incidence of 8 to 12 per million. 1 2 Due to its short survival, it is considered a hematologic emergency. Life-saving chemotherapy, bone marrow transplantation, and monoclonal antibodies are treatment options for this hematologic malignancy.

The prevalence of ATTR amyloidosis is 10 to 16% in older patients with heart failure with preserved ejection fraction or severe aortic stenosis. 3 4 5 Subtypes are identified based on mutation status as wild-type (ATTRwt) and hereditary. ATTRwt amyloidosis is more common. In recent years, there have been some developments in treatment options, including suppressing transthyretin production (liver transplantation, patisiran, inotersen), stabilizing amyloid fibrils (tafamidis, NSAID [nonsteroidal anti-inflammatory drug] diflunisal), and destroying already existing fibrils (doxycycline, tauroursodeoxycholic acid, and monoclonal antibodies). 6

Echocardiography (ECHO) is a noninvasive, accessible method to assess the functional and structural status of the myocardium. Ventricle and valvular thickening, biatrial enlargement, restrictive filling pattern with generally preserved systolic function, and low voltage are commonly detected in CA.

Laboratory tests could be used to differentiate these two forms of amyloidosis and serve as prognostic biomarkers. Cardiac magnetic resonance imaging (CMR) provides important clues for distinguishing amyloidosis in hypertrophic hearts. T1 values, extracellular volume, and late gadolinium enhancement are significant parameters in both the differential diagnosis and prognostic assessment.

Endomyocardial biopsy was accepted as the gold standard in the diagnosis of CA. However, it is an invasive procedure and could yield false negatives due to patchy involvement.

[ ^99m^ Tc]Tc scintigraphy with bone-seeking radiotracers such as pyrophosphate (PYP), 3,3-diphosphono-1,2-propanodicarboxylic acid (DPD), and hydroxymethylene diphosphonate is a reliable diagnostic tool with high sensitivity and specificity in CA. 7 Cases with positive scintigraphy with bone-seeking radiotracers along with negative serum free light chain (FLC) in serum/and urine immunofixation electrophoresis are diagnosed with ATTR CA.

Beyond the diagnostic power, recent data highlighted the prognostic potential of Tc-PYP scintigraphy in CA. 8 However, there is still disparity about the prognostic value of [ ^99m^ Tc]Tc-PYP scintigraphy in the literature. In this single-center retrospective study, we aimed to describe our scintigraphic findings and determine the prognostic value of scintigraphy.

## Materials and Methods

### Study Population

The study cohort consisted of patients who were referred from a tertiary cardiac center between September 2020 and December 2023 for the suspicion of CA involvement, based on clinical, laboratory, and imaging findings. All patients had heart failure with preserved ejection fraction or diastolic dysfunction, along with a thickened myocardial wall on ECHO, in the absence of other loading conditions. There was at least one finding in cardiac imaging methods raising suspicion of amyloidosis in the study population. Simultaneously with scintigraphy, the serum FLC level and the immunofixation electrophoresis of serum and urine were measured in the cohort to eliminate AL CA. In suitable cases, AL CA diagnosis was confirmed histopathologically.

All participants underwent [ ^99m^ Tc]Tc-PYP scintigraphy and images were retrospectively evaluated by two experienced nuclear medicine specialists. Vital status was ascertained through telephone contact and/or by contacting referring physicians. Overall survival (OS) was defined as the time from randomization to death from any cause. Cases with dead were expressed as deceased cases.

### Imaging Procedure

After the intravenous injection of 740 MBq [ ^99m^ Tc]Tc-PYP, 1-hour and 3-hour anterior–posterior planar and single-photon computed tomography/computed tomography (SPECT/CT) images (NM 860, GE HealthCare, United States) were obtained over the thoracic region using a 140 keV 15 to 20% energy window, a 128 × 128 matrix, and a low-energy general-purpose collimator according to the guidelines. 9 Low-dose CT was used for attenuation correction.

### Imaging Analysis

For quantitative evaluation, the heart-to-contralateral lung (H/CL) ratio was calculated on the anterior planar images as the total counts in a region of interest (ROI) over the heart divided by the identical size of ROI over the contralateral chest. A ratio greater than 1.5 at 1 hour and greater than 1.3 at 3 hours is accepted as positive. Ratios lower than 1 are accepted as negative, and those between 1 and 1.5/1.3 are considered equivocal. 9 Cases with equivocal results were included in the analysis according to biopsy results, if available.

**Fig. 1:**
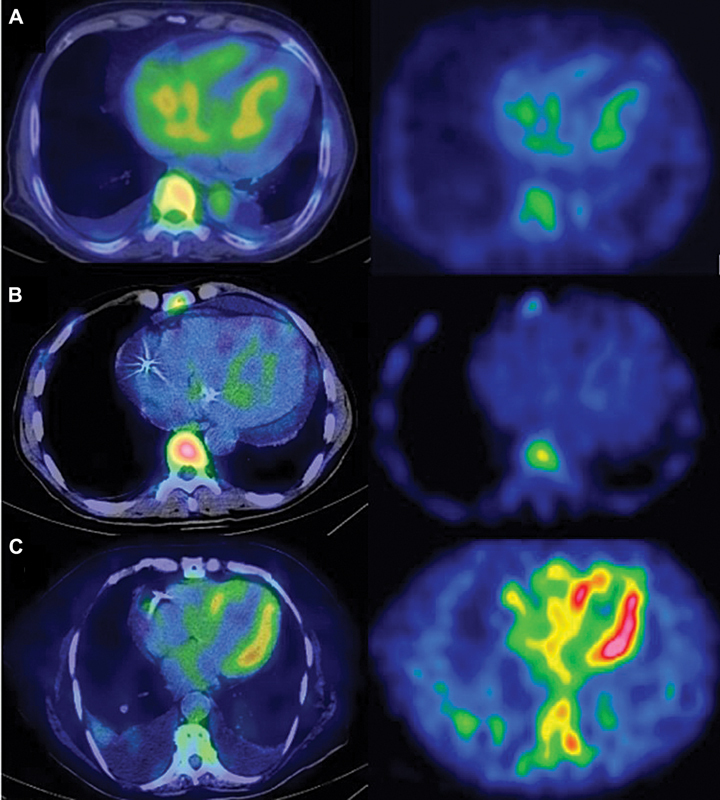
[ ^99m^ Tc]Tc-PYP scintigraphy semiquantitative scoring in 3-hour images. Row ( **A** ): score 0/negative; row ( **B** ): score 1/equivocal; row ( **C** ): score 2/positive. Left column: SPECT/CT fusion images; right column: SPECT images. SPECT/CT, single-photon computed tomography/computed tomography.

The uptake of [ ^99m^ Tc]Tc-PYP in the myocardium was evaluated using a semiquantitative visual score on SPECT/CT images (grade 0: no myocardial uptake; grade 1: myocardial uptake < Rib uptake; grade 2: myocardial uptake = Rib uptake; grade 3: myocardial uptake > Rib uptake). In terms of ATTR CA, score 0 (grade 0) is classified as negative, score 1 (grade 1) is equivocal, and score 2 (grades 2 and 3) is positive ( Fig. 1 ). Myocardial uptake in the apex, which could be related to previous infarction, was not accepted as positive for CA.

AL CA was defined by bone marrow biopsy-proven congophilic deposits, or histological documentation of congophilic deposits in at least one noncardiac organ and ECHO findings of restrictive cardiomyopathy, defined as left ventricle (LV) wall of 12 mm or greater in thickness without another etiological factor of LV hypertrophy. Positive PYP scintigraphy, along with the absence of serum FLC in serum/and urine immunofixation electrophoresis and the presence of ECHO findings related to heart failure with preserved ejection fraction, was accepted as ATTR CA. 10

The median follow-up time was 386 days. The cardiac [ ^99m^ Tc]Tc-PYP uptake and scintigraphic findings were correlated with follow-up data.

### Statistical Analysis

Statistical analyses were performed using SPSS software, version 15. Descriptive analyses were presented using the median (range). The Mann–Whitney U-test and Kruskal–Wallis test were conducted to compare nonparametric ordinal and nominal values between groups. The Spearman test was used to investigate the correlation coefficients and their significance between the variables.

We compared Kaplan–Meier curves for all time-to-event outcome measures with the standard log-rank test. A *p* -value of less than 0.05 was considered significant.

## Results

A total of 268 cases (147 females, 121 males) were included in this study. The median age was 65 (range: 21–90). Median proBNP and troponin T values were 2,198 pg/mL and 96 ng/mL (range: 10–37,905 pg/mL and 0.008–1,762 ng/mL), respectively.

Among the 268 patients, 12 (4.5%) were diagnosed with ATTR CA, and 19 (7.1%) with AL CA. According to genetic test results, only 2 (17%) of ATTR CA cases were mutation-positive; others were accepted as wild-type. AL diagnoses were confirmed with biopsy in eight cases, while the rest were AL-suspected according to laboratory test results. All AL CA cases were referred to the hematology department for planning life-saving chemotherapy.

**Fig. 2:**
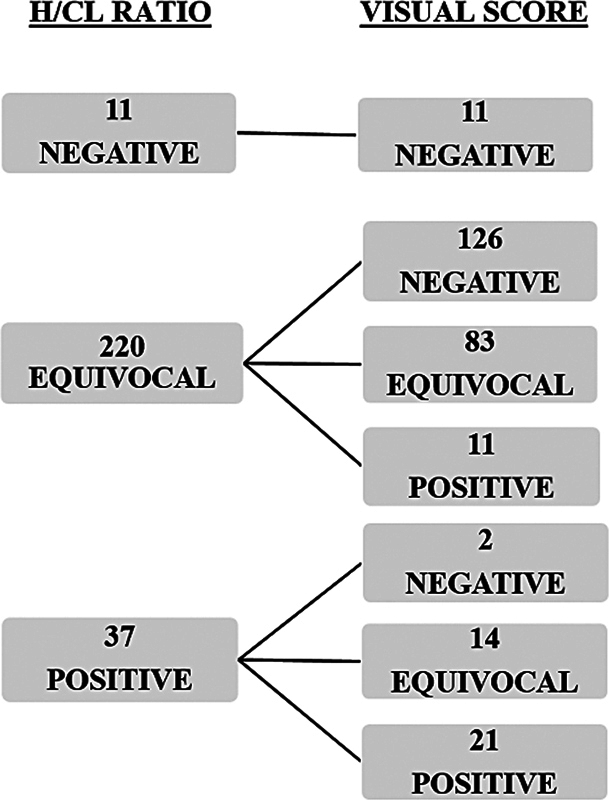
Quantitative (H/CL ratio) and semiquantitative (visual score) results of all cases in [ ^99m^ Tc]Tc-PYP scintigraphy for 3-hour images. H/CL, heart/contralateral lung.

**Fig. 3:**
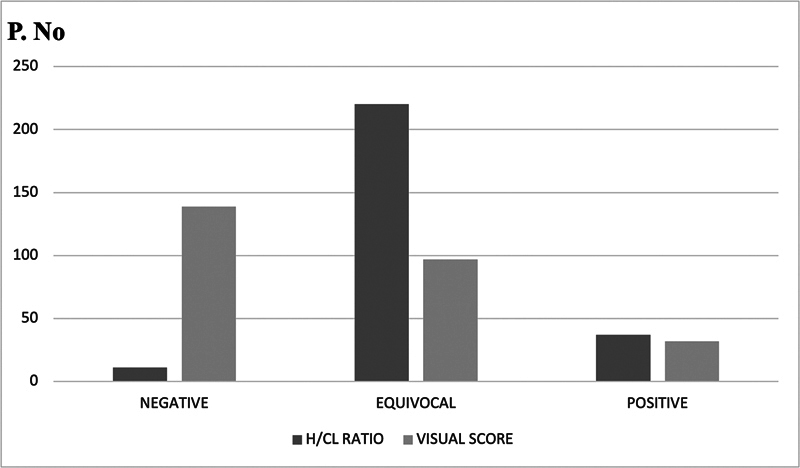
Comparative graphical representation of quantitative and semiquantitative results. H/CL, heart/contralateral lung.

Quantitative and semiquantitative results of [ ^99m^ Tc]Tc-PYP scintigraphy are shown in Fig. 2 . The 1-hour and 3-hour H/CL ratios were highly positively correlated ( *r* = 0.80, *p* < 0.001) with each other. A significant but moderately correlated relationship was detected between quantitative and semiquantitative results ( *r* = 0.52, *p* < 0.001). Compared with the H/CL ratio, semiquantitative assessment reduced the equivocal result ratio from 82 to 37% ( Fig. 3 ).

First hour H/CL ratio median values were 1.7 (range: 1.4–2.1) in ATTR CA cases and 1.2 (range: 0.9–1.6) in AL CA. We observed significantly higher PYP uptake in ATTR patients compared with AL CA ( *p* < 0.001).

The median follow-up was 386 days (range: 10–1,075 days). During the follow-up term, 1 (8.3%) of ATTR CA cases and 8 (42.1%) of AL CA cases died. Fifteen cases (6%) with nonamyloid heart failure with preserved ejection fraction (non-CA deceased cases) also died. Median survival was 22 days for ATTR CA deceased cases, 201 days (range: 26–626 days) for AL CA deceased cases, and 105 days (range: 10–313 days) for non-CA deceased cases.

The H/CL ratio median value was 1.3 (range: 1.1–1.5) in non-CA nonsurvivors. Both quantitative and semiquantitative results were significantly different among ATTR CA, AL CA, non-CA deceased, and others ( *p* < 0.001). In ATTR CA cases, PYP uptake was more prominent than others ( Table 1 ). Semiquantitative and quantitative results are also shown in Table 1 for the deceased case groups. As only one patient with ATTR CA died on follow-up, survival could not be compared between ATTR CA cases and other groups.

**Table 1:** Quantitative and semiquantitative results of CA diagnosed and deceased cases

		H/CL ratio, median (range) ( *n* )	Visual score
**ATTR CA (all cases)** *n* = 12	**Positive**	1.7 (1.5–2.1) (11)	12
	**Equivocal**	1.4 (1)	0
	**Negative**	(0)	0
**ATTR CA (deceased cases)** *n* = 1	**Positive**	1.7 (1)	1
	**Equivocal**	(0)	0
	**Negative**	(0)	0
**AL CA (all cases)** *n* = 19	**Positive**	1.6 (1.5–1.6) (2)	1
	**Equivocal**	1.2 (0.9–1.4) (17)	10
	**Negative**	(0)	8
**AL CA (deceased cases)** *n* = 8	**Positive**	1.5 (1)	0
	**Equivocal**	1.3 (0.9–1.4) (7)	6
	**Negative**	(0)	2
**Non-CA deceased cases** *n* = 15	**Positive**	1.5 (1.5–1.5) (2)	1
	**Equivocal**	1.3 (1.1–1.4) (13)	8
	**Negative**	(0)	6

Abbreviations: AL, light chain; ATTR, transthyretin; CA, cardiac amyloidosis; H/CL, heart/contralateral lung.

**Fig. 4:**
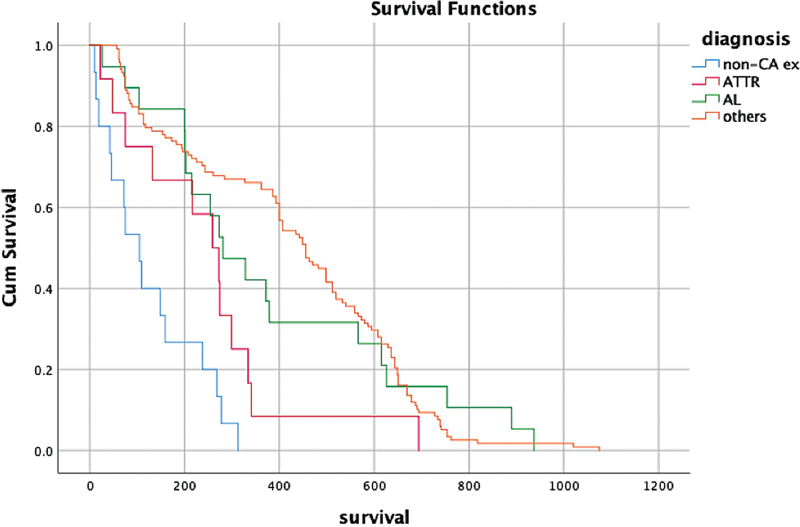
Kaplan–Meier survival graphic of each group. AL, light chain, ATTR, transthyretin, CA, cardiac amyloidosis.

The median OS was 261 days (range: 22–694) for ATTR CA patients, 281 days (range: 26–937) for AL CA patients, 105 days (range: 10–313) for non-CA deceased cases, and 456 days (range: 57–1,075) for non-CA survivors. We noticed that OS was significantly lower for non-CA deceased cases ( *p* < 0.001; Fig. 4 ). We observed that visual scores and H/CL ratios significantly overlapped between AL CA patients and non-CA diagnosed nonsurvivors.

A subgroup analysis was performed by categorizing the patients with CA and patients who died during the follow-up term as one group and the survivors without CA as the other. Then, possible prognostic factors, such as age, gender, 1-hour and 3-hour H/CL ratios, semiquantitative results, proBNP, and troponin T values, were analyzed between groups. Among these parameters, 1-hour and 3-hour H/CL ratios, semiquantitative results, proBNP, and troponin T values were significantly higher in the group consisting of CA and nonsurvivors than survivors ( Table 2 ).

**Table 2:** Demographic and scintigraphic data of each group

CA and/or deceased cases, *n* = 46		Nondeceased cases, *n* = 222	*p* -Value
**Age** , median (range)	68 (37–90)	63 (21–85)	0.03
**Gender**	25 male	113 male	<0.001
**1-hour H/CL ratio** , median (range)	1.3 (0.9–2.1)	1.2 (0.8–1.5)	<0.001
**3-hour H/CL ratio** , median (range)	1.3 (0.9–2.1)	1.2 (0.8–1.6)	<0.001
**Visual scores**			
Negative	14	125	<0.001
Equivocal	18	79	
Positive	14	18	
**ProBNP** , pg/mL, median (range)	5,666 (363–37,905)	1,562 (10–30,935)	<0.001
**Troponin T** , ng/mL, median (range)	46 (13–304)	22.5 (0.008–1,762)	<0.001

Abbreviations: CA, cardiac amyloidosis; H/CL, heart/contralateral lung.

When myocardial PYP uptake was analyzed for prognostication within the whole cohort, we observed that OS deteriorated with increasing LV PYP uptake. We observed that the 1-hour H/CL ratios and quantitative results were significantly correlated negatively with OS; however, the correlation degree was low ( *r* = −0.16, *p* = 0.04). When visual scoring was tested for OS prediction, we observed worsening OS with increasing visual score. The median was 960 days (range: 883–1,036 days) for patients with score 0, whereas OS was 806 days (range: 709–904 days) and 618 days (range: 519–717 days) for patients with scores 1 and 2, respectively. However, the difference did not reach statistical significance ( *p* = 0.18).

When subgroup analyses were performed among AL CA cases, we observed that a 1-hour H/CL ratio cutoff of 1.55 seems to be very sensitive (100%) and specific (91%) in predicting short survival. Although a trend toward significance was observed, it was not statistically significant (area under the curve: 0.60, *p* = 0.46).

## Discussion

Amyloidosis with cardiac involvement was, until recently, considered a rare disease. With improvements in diagnostic modalities and increasing awareness, it was revealed that it is not as rare as thought before. Treatment modalities for the main CA forms, namely ATTR CA and AL CA, caused by different misfolded proteins with different origins, are different. 11 Considering the short survival of these two entities, early diagnosis becomes quite important.

In the past, invasive cardiac biopsy was considered the gold standard in diagnosis. Since the high sensitivity (>99%) and specificity (86%) of scintigraphy using [ ^99m^ Tc]Tc-labeled bone-seeking agents have been proven, the clinical diagnostic algorithm has changed. 7 12 13 The pathophysiology of cardiac uptake of bone-seeking radiopharmaceuticals is not well known. The presence of microcalcification in ATTR but not in AL amyloidosis could be a possible explanation for the sensitivity of these tracers in ATTR CA. 14 Consistent with the literature, we revealed that cardiac uptake intensity was significantly higher in ATTR CA than in others. 7 12 13

Similar to our results, Coskun et al found a strong positive correlation between 1-hour and 3-hour H/CL ratios. 15 They found that SPECT/CT imaging reduced the rate of equivocal results by 65%, which was calculated as 55% in our study (82–37%). The difference may be attributed to the difference in labeling procedures of the PYP kit or different experiences of the readers.

Patients with positive clinic, ECHO, and/or CMR findings, along with positive [ ^99m^ Tc]Tc-PYP scintigraphy results, are diagnosed with CA ATTR. 16 Due to the unfavorable prognosis of CA, if there is clinical suspicion of CA, PYP scintigraphy and serum and urine immunofixation and serum FLC studies are recommended to be performed simultaneously. Although, according to current guidelines, grade 2–3 radiopharmaceutical uptake is considered positive for ATTR CA, it is well-known that it could be seen in AL CA. 9 In our study, a significant portion of AL CA cases (58%) showed a varying degree of increased activity even in SPECT images. This finding proves once again how important laboratory tests are in addition to scintigraphic data in the diagnosis of AL CA. Since AL CA is an oncological emergency, scintigraphic findings should always be interpreted in conjunction with laboratory test results to exclude AL amyloidosis. Moreover, some of these cases could be the result of a double pathology. ATTR CA, along with AL CA, could be an explanation for the higher PYP uptake. However, in these cases, diagnosis of ATTR CA is not easy, even with endomyocardial biopsy.

In the largest single-center study with 292 AL CA cases with ^99m^ Tc-DPD scintigraphy, 39% of AL CA cases showed cardiac uptake in scintigraphy images. 17 In these cases, the uptake level was not as prominent as in ATTR CA cases. Our discordant results may be due to the use of different radiopharmaceuticals (DPD vs. PYP), patients' different systolic function, and/or differences in specialists' experiences. Quarta et al also reported that only cardiac laboratory test results and serum bilirubin levels were associated with worse survival. 17 The working group concludes that the presence of cardiac [ ^99m^ Tc]Tc-DPD uptake showed a trend to worse survival, but it was not significant ( *p* = 0.056). Similarly, we did not find any significant correlation between survival and any scintigraphic findings in the subgroup analysis of AL CA patients. Although in our cohort, a better OS of AL was observed when compared with ATTR CA, we think this comparison did not reflect the truth. As we stated earlier, some of the AL cases were not precisely diagnosed histopathologically.

A previously published multicenter study showed that [ ^99m^ Tc]Tc-PYP uptake can accurately differentiate patients with poorer survival. 8 Castano et al reported that H/CL greater than 1.6 was associated with shorter survival in patients with ATTR CA. In our study population, treatment with tafamidis was started as early as when the patients were diagnosed with ATTR CA. In the cohort, only one ATTR CA case died. The positive effect of tafamidis on survival in ATTR CA cases is an important development in the treatment. The early prescription of tafamidis may have contributed to prognosis in ATTR CA patients. 18

We consider that the most remarkable result was the significantly lower survival of non-ATTR CA cases in our study population. We hypothesized that there may have been ATTR CA patients who did not have pronounced PYP uptake among the patients with H/CL ratios ranging between 1.1 and 1.5. It is known that up to 28.5% of ATTR CA cases may be represented by low H/CL ratios. 19 As endomyocardial biopsy was not performed for all patients, we consider that cases with early involvement of ATTR CA may have been underdiagnosed. It is possible that some of the 15 non-CA deceased cases were nondiagnosed ATTR CA.

Even though we cannot perform a survival analysis among our ATTR CA patients, we consider that the inverse correlation observed between PYP uptake and OS in the cohort highlights the need for further evaluation and close follow-up for cardiac events for patients who have clinical suspicion of CA, even though the scintigraphy findings did not suggest a clear diagnosis of ATTR CA.

There are some limitations of our study. The main limitation of this study was the retrospective design. Second, this study was a single-center study with a relatively limited number of positive cases. Prognostic outcomes of this study should be confirmed with larger cohorts. We could not analyze the prognostic value of PYP uptake among a small group of ATTR cases. We believe that with more power, better results for survival analysis and cut-off values would have been defined. Third, we consider that a longer period of follow-up in the population with less pronounced H/CL is required to better test whether meaningful changes in the H/CL ratio occur and whether [ ^99m^ Tc]Tc-PYP can identify patients with early involvement. Finally, the exact etiology of cardiac deaths among the non-CA subgroup remained unexplained. The indication of endomyocardial biopsy may further be questioned in patients with high clinical suspicion of CA with negative or equivocal findings in PYP scan to prevent the underestimation of early ATTR involvement.

## Conclusion

[ ^99m^ Tc]Tc-PYP cardiac imaging is highly sensitive and specific for detecting ATTR CA and differentiating from AL CA. A higher H/CL ratio was observed in nonsurvivors irrespective of a definite ATTR diagnosis. Patients with high uptake (H/CL ratios between 1.3 and 1.5) but not diagnosed with ATTR should also be closely monitored.
